# Predictors of intention to receive cervical cancer screening among commercial sex workers in Gondar city, northwest Ethiopia: application of the theory of planned behavior

**DOI:** 10.1186/s12905-022-02055-8

**Published:** 2022-11-21

**Authors:** Habitu Birhan Eshetu, Kegnie Shitu, Simegnew Handebo

**Affiliations:** 1 Department of Health Promotion and Health Behavior, Institute of Public Health, College of Medicine and Health Sciences, University of Gondar, PO.Box.196, Gondar, Ethiopia; 2 School of Public Health, Saint Paul’s Hospital Millennium Medical College, Addis Ababa, Ethiopia

**Keywords:** Intention, Cervical cancer screening, Commercial sex workers, Theory of planned behavior, Gondar

## Abstract

**Background:**

Cervical cancer is a global public health problem & is the fourth leading cause of cancer morbidity and mortality. Abnormal cervical lesion is common in commercial sex workers and is at a higher risk of developing cervical cancer due to multiple sexual partners besides other factors. Intention is an important predictor of behavior and is an initiative to transform their desire into action. Therefore, this study aimed to assess the predictors of intention to receive cervical cancer screening among commercial sex workers in Gondar city, northwest Ethiopia.

**Methods:**

A community-based cross-sectional study was conducted from March 27 to May 25, 2021, in Gondar city, northwest Ethiopia. A total of 425 commercial sex workers selected using convenience sampling techniques were included in the study. Linear regression with robust standard errors was carried out to identify predictors of intention to receive cervical cancer screening. A 95% confidence interval and a *p*-value of less than 0.05 were used to declare statistical significance.

**Results:**

A total of 393 commercial sex workers participated in the study with a response rate of 92.4%. The mean age of the participants was 27.68 ± 6.62. The median (interquartile range) of intention was 4 (3–4.25). The theory of planned behaviour variables explained 38.51% of the variance in intention to receive cervical cancer screening. Direct subjective norm (β = 0.09), 95% CI (0.05, 0.13)), direct Attitude (β = 0.09, 95% CI (0.04, 0.13)), past behaviour (β = 0.27; 95% CI (0.09, 0.46), and positive HIV status (β = 0.26; 95% CI (0.06, 0.46) were significant predictors of intention.

**Conclusions:**

Commercial sex workers' intention to undergo cervical cancer screening was high. The theory of planned behavior showed adequate utility in predicting commercial sex workers’ intention to receive cervical cancer screening. Participant’s attitudes, subjective norm, past behavior, and positive HIV status were important factors affecting their intention to receive cervical cancer screening. Thus, interventions aimed at enhancing commercial sex workers’ cervical cancer screening behavior should target creating positive social pressure and attitudinal change towards cervical cancer screening.

## Background

Cervical cancer is one of the commonest diseases globally, in which malignant cells form in the woman's reproductive organ in the tissues of the cervix. Human Papilloma Virus (HPV) is the commonest cause accounting for 90% of cervical cancer [1]. Cervical cancer is the fourth most prevalent cancer in women, around 570 000 cases have been diagnosed worldwide. About 311 000 have died of cervical cancer, of which 90% were in low-and middle-income countries (LMICs) [2, 3]. One in 65 women developed cervical cancer over a lifespan globally, and the chances were the highest in LMICs countries (1 in 40) and the lowest in high-income countries(HICs) (1 in 106) [4]. In sub-Saharan Africa, the incidence of cervical cancer was high as compared to high-income countries 23.2% and 2.1%, respectively [5]. In Ethiopia, cervical cancer is the second most common cancer and cause of death among women. In 2020, 7445 new cervical cancer cases and 5338 deaths due to cervical cancer is reported [6].

HPV infection is highest among sex workers [7–9].In addition, they are at higher risk of getting HIV/AIDS as compared to the general population [10]. The complication of cervical cancer increases as it advances [11, 12]. The long-term consequences of cervical cancer at the individual level include a change in sexual life, urinary difficulties, change in bowel movement, infertility, bone involvement, and distance metastasizing to the liver and lung [13]. Moreover, it also leads to cognitive impairment, anxiety, and depression [14, 15]. The health and medical cost are more than 10 million $ and the social cost ( including travel costs, indirect and non-medical cost including loss of productivity is more than 82 million$ [16]. In Ethiopia, the average inpatient and outpatient cost of cervical cancer is $404.4 and $407.2 per patient respectively [17].

Screening is very important to effectively reduce the incidence of cervical cancer and mortality [18, 19]. The precancerous stage of cervical cancer is long and the survival of the patients in this stage is almost 100% if diagnosed and treated. Regular screening can detect more than 90% of cervical cancer in its early stage. However, in Ethiopia, the coverage of cervical cancer screening is 0.6% for women aged between 18–69, 1.6% in urban and 0.4% in rural [20, 21].

The theory of planned behavior (TPB) was proposed by Icek Ajzen in 1985 and since then has been used for different health-related research [22]. Intention to receive cervical cancer screening varies across different studies. According to the theory of planned behavior, a person’s behavior is influenced directly by intention and indirectly by attitudes toward the behavior, subjective norms, and perceived behavioral control [23]. A meta-analysis revealed that the TPB accounted for 39% of the variance in intentions and 27% of the variance in behavior across a wide range of behaviors [24]. Evidence also supports that TPB is valuable in predicting cervical cancer screening intention [25–28]. For example, in a study of America Latina, the variance explained in intentions to receive cervical cancer screening was, (65%), and (27.6%)[25, 29], in Canada 39% [30], in Thailand 28.1% [31], and in Iran revealed 57.4% [32]. Other factors which predict cervical cancer screening include monthly income, education level, age, and knowledge level [27, 32–34]. As far as the researcher’s knowledge, there is no study done on commercial sex workers' intention to receive cervical cancer screening. Therefore, this study aimed to assess the intention to receive cervical cancer screening among commercial sex workers and its predictors among commercial sex workers in Gondar city, northwest Ethiopia.

## Methods

### Study area and design

The study was conducted in Gondar city administration, which is located about 727 km away from Addis Ababa, the capital city of Ethiopia, and 180 km away from Bahir Dar the capital city of Amhara Regional State. The city has a total population of 432,191 people with 256,041 people whose age is between 18 and 65 years old. There are 51 private and public health facilities in the city administration. Of these 43 health institutions are private, with one private general hospital and one public specialized referral hospital [35]. According to the social affairs of each sub-city report, there were above 2431 commercial sex workers in Gondar city. A community-based cross-sectional study was conducted from March 27 to May 25, 2021.

### Participants

In this study, all commercial sex workers living in Gondar were the source population. Those Commercial sex workers, who had undergone a total hysterectomy, know they had confirmed cancer of the cervix, and screened for cervical cancer in the past four years were excluded from this study.

### Sample size determination and sampling procedure

The sample size was calculated using a single population mean formula by considering the following assumptions: Z α/_2_ = 1.96 standard score corresponding to 95% CI, margin of error (d = 0.4), and Mean = 14.52 (_δ_ = 4.01) (the mean score and standard deviations of intention to receive cervical cancer screening service Jimma) [28]_._ Following the formula and taking 10% of non-response rate, the final sample size was 425. Four sub-cities (Maraki, Jantekel, Fasil, and Arada) were selected randomly. The sample size was proportional allocated to selected sub-cities based on the number of commercial sex workers registered in each social affairs. The study participants were recruited from hotels, brothels, and their houses in the selected sub-cities using convenience sampling technique. The participants were recruited until the required sample size was reached.

### Study variables

The outcome variable of this study was intention to receive cervical cancer screening. The Independent variable was socio-demographic variables (age, marital status, family size, educational status, religion, income), Past experience of cervical cancer screening, knowledge about cervical cancer screening, history of STI, HIV status, and Theory of planned behaviour variables direct attitude, indirect attitude, direct subjective norm, indirect subjective norm, direct perceived behavioural control, indirect perceived behavioural control.

### Data collection

The data were collected after a structured questionnaire was developed in English from relevant pieces of literature [28, 36, 37], and an elicitation study. Then, the questionnaire was translated to Amharic by two senior health education and behavioral science instructors. The Amharic questionnaire was re-translated back to English by other senior health education and behavioral science instructors to check the consistency of translation. The tool was validated in terms of face and content validity by seven experts. The Amharic version questionnaire was used for data collection. Three diploma-holder nurses were assigned to collect data and one Bachelor of Science in public health participated for supervision. The training was given for two consecutive days for data collectors and the supervisor. The training included a discussion about the objective of the study, the technique of data collection, the content of the questionnaire, issue of confidentiality of the participants. Before the actual data collection, the questionnaires were pre-tested on 5% [21] of the total sample size. Based on the findings of the pre-test, there were modifications of vague questions and concepts on the tool before applying it to actual study participants.

### Measurements

#### Intention

The perceived likelihood of being screened for cervical cancer in the coming one year. It was measured with four items having five points Likert scale each. The response scale ranged from “very unlikely” (1) to “very likely” (5). The composite score ranged from four to twenty and the high score indicated high intention [23]. The reliability test for Cronbach’s alpha was 0.93.

#### Direct attitude (DAT)

Individuals' feelings and beliefs about cervical cancer screening. It was measured with four items having with five-point Likert scale each. The composite score ranges from four to twenty and a high score indicates a high attitude [23]. The reliability test for Cronbach’s alpha was 0.91.

#### Direct subjective norm (DSN)

Individual’s belief about whether most people approve or disapprove the cervical cancer screening. It was measured with four items having with five-point Likert scale each. The composite score ranges from four to twenty the higher score indicates high social influence toward cervical cancer screening [23]. The reliability test for Cronbach’s alpha was 0.87.

#### Direct perceived behavioural control (DPBC)

The perceived ability of an individual to control factors, which influenced cervical cancer screening. It was measured with four items having with five-point Likert scale. The composite score ranges from four to twenty and the higher score indicated higher perceived ability of individuals to control factors [38]. The reliability test for Cronbach’s alpha was 0.68.

#### Indirect attitude (IATT)

It is composed of behavioral belief i.e., one’s belief about the likely outcome of being screened for cervical cancer and outcome evaluation i.e. one’s judgmental evaluation of the outcome of the behavior. It was assessed by five items, for behavioral belief measuring and five items for outcome evaluation measurement. The final value of the variable was produced by computing the product sum of the sub-components (behavioral belief (b_i_) and outcome evaluation (e_i_) [23].

#### Indirect subjective norm(ISN)

derived from list referents who would approve of them to screen, such as volunteers, health workers, and HEW (health extension worker) and friends are the most influential referent others. Then four normative belief measuring items and four motivation to comply items will be prepared. The final value was produced by the product sum of normative belief (n_i_) and motivation to comply (m_i_)[23].

#### Indirect perceived behavioural control (IPBC)

Is controlling factors that facilitate or hinder to have cervical cancer screening and perceived power. Then five control belief measuring items and five perceived power items was prepared, the final value is directly proportional to the composite score derived by summing the products of control belief strength (ci) times perceived power (pi) overall accessible control factors [37, 39].

#### Knowledge

CSWs (commercial sex workers) knowledge regarding cervical cancer screening. It was measured with 13 items, Participants overall knowledge was categorized using modified Bloom’s cut-off point, as good if the score was between 80 and 100% (10.4–13 points), moderate if the score was between 50 and 79% (6.5–10.3 points), and poor if the score was less than 50% (< 6.5 points) [40].

### Data processing and analysis

The collected data was cleaned and entered into Epi data version 4.6 and it was exported to Stata version 16 for analysis. Data cleaning, recoding, and checking for missing values were made. Normality assumptions of the dependent variable was checked using skewness and kurtosis test (-0.88 and 2.9 respectively) and it was moderately skewed distributed. But skewness between + 2 and-2 is considered normal when the sample size is greater than 300 [41]. As a result, simple linear regression was employed to examine the association of each independent variable with intention to receive cervical cancer screening. Variables with a *p*-value less than 0.25 in simple linear regression were entered into multiple linear regression. Linearity, normality, constant variance, outliers, and multicollinearity assumptions were checked.

Linearity assumptions were checked using a scatter plot of the dependent and independent variables for simple linear regression, and between standardized residuals versus the predicted values from the regression analysis for multiple linear regression. Objective test like curve estimation was also checked to examine the relationship of the variables. There was a linear relationship between dependent and independent variables. Test of homoscedasticity using Cameron & Trivedi's decomposition of IM-test with the number of predictors was conducted and it shows insignificant. Multicollinearity assumptions were tested by the variance inflation factor (VIF) and the value of all variables was below five. The assumption of an outlier was tested using Cook’s D and it shows the presence of outliers. The presence of outliers makes the usual ordinarily least square regression useless in predicting the confidence interval or the standard error correctly. Owing to this the alternative methods of robust regression were held to account for the assumptions. R-square was used to assess the variation in intention to receive cervical cancer screening explained by the independent variables. An unstandardized β coefficient was used to interpret the effect of the independent variable on the intention to receive cervical cancer screening.

## Results

### Socio-demographic characteristics

A total of 393 respondents participated with a response rate of 92.4%. The mean age of participants with standard deviation was (27.68 ± 6.62) and ranged from 18 to 46. The mean length spent in sex work with a standard deviation was (3.9 years SD ± 3.4). Regarding their educational status, 138(35.11%) have no formal education (Table 1).

**Table 1:** Socio-demographic characteristics of commercial sex workers in Gondar city, 2021, (*n* = 393)

Variables		Frequency	Percent (%)
Age in years (mean ± SD)		27.68 ± 6.62	
Residence before sex work	Urban	148	(37.76)
	Rural	245	(62.34)
Marital status	Never married	191	(48.60)
	Ever married	202	(51.40)
Educational status	No formal education	194	(49.36)
	primary education	131	(33.33)
	secondary and above	68	(17.30)
Religion	Orthodox	359	(91.35)
	Muslim	31	(7.89)
	Jewish	3	(0.76)
Place of sex work establishment	hotel	89	(22.65)
	Brothel	78	(19.85)
	rented house	194	(49.36)
	Street	21	(5.38)
	Other	9	(2.76)
Family they help	yes	198	(50.4)
	No	195	(49.6)
Length spent in sex work(mean ± SD) year		(3.9 ± 3.4)	
Family ever had cervical cancer		8	(1.91)

### Magnitude of intention

The normality of intention to receive cervical cancer was checked using skewness values and was negatively skewed from normality. The median intention of the respondents was 4 (interquartile ranges (IQR) 3–4.25).

### Knowledge on cervical cancer screening

The mean knowledge score about cervical cancer of respondents was found to be 5.02 (± 1.34) and it ranged from 0 to 13. The majority of the study participants had poor knowledge about cervical cancer screening 343 (87.28%) based on modified bloom’s classification. Regarding the cause of cervical cancer 159 (40.46%) report multiple sexual partners (Table 2).

**Table 2:** Knowledge of commercial sex workers about cervical cancer in Gondar city, 2021, (*n* = 393)

Cause of cervical cancer	Correct, n (%)	Incorrect, n(%)
Multiple sexual partners	159(40.46)	234(59.54)
Early sexual intercourse	72 (18.32)	321(81.68)
HPV infection	26 (6.62)	367 (93.38)
Cigarette smoking	28(7.12)	365(92.88)
**Sign and symptoms**		
Vaginal bleeding	62 (15.78)	331 (84.22)
Foul smelling vaginal discharge	126 (32.06)	267 (67.94)
bleeding during and after intercourse	45(11.45)	348(88.55)
Pain during intercourse	183 (46.56)	210 (53.44)
Cervical cancer is preventable disease	356 (90.59)	37 (9.41)
Do you think cervical cancer can be screened	369 (93.89)	24 (6.11)
Appropriate age group to be screened (25–65 years)	177 (45.04)	216 (54.96)
Interval to be screened (every five years)	116 (29.52)	277 (70.48)
Better cured if early treated	162(41.22)	231 (58.78)

*N* Number

### Theory of planned behavior variables

The mean score of all variables of TPB of the respondents was above the neutral. The mean score of direct attitude was 4.04 ± 0.72 and the direct subjective norm mean score was 3.73 ± 0.73 (Table 3).

**Table 3:** Descriptive statistics of TPB variables among CSWs in Gondar city, 2021, (*n* = 393)

Variables	Mean	SD	Min	Max
Direct attitude	4.04	0.72	1	5
Direct subjective norm	3.73	0.73	1	5
Direct perceived behavioral control	3.76	0.65	1.25	5
Indirect attitude	84.77	25.46	8	125
Indirect subjective norm	65.22	21.24	12	100
Indirect perceived behavioral control	60.78	31.48	6	125

*SD* Standard deviation, *Min* Minimum, *Max* Maximum

### Correlation among TPB constructs

Spearman rank correlation analysis was done to explore the relationship among the theory of planned behavior variables. Accordingly, all TPB variables showed a positive and significant correlation with intention at a *p*-value of < 0.05. All the TPB variables had a strong relationship except indirect perceived behavioural control, which showed a weak relationship with intention. All indirect measures of TPB variables had positive and strong correlations with their respective direct measures (Table 4).

**Table 4:** Spearman correlation of TPB variables among CSWs in Gondar city, 2021, (*n* = 393)

Variables	Intention	DAT	DSN	DPBC	IATT	ISN	IPBC
Intention	1						
Direct attitude(DAT)	0.53^a^	1					
Direct subjective norm(DSN)	0.56^a^	0.62^a^	1				
Direct perceived behavioral control(DPBC)	0.54^a^	0.59^a^	0.65^a^	1			
Indirect attitude(IATT)	0.57^a^	0.69^a^	0.64^a^	0.68^a^	1		
Indirect subjective norm(ISN)	0.56^a^	0.60^a^	0.66^a^	0.53^a^	0.67^a^	1	
Indirect perceived behavioral control(IPBC)	0.38^a^	0.24^a^	0.37^a^	0.45^a^	0.27^a^	0.29*	1

^a^Correlation is significant at 0.05(2 tailed)

### Predictors of intention to receive cervical cancer screening

Socio-demographic (age, educational status), past experience of cervical cancer screening, HIV status, history of STI, and all the direct measures of TPB variables were a candidate for multiple linear regression at *p*-value < 0.25. In multiple linear regression variables statistically significant at a 5% level of significance were direct attitude, direct subjective norm, positive HIV status, and past screening experience. The standardized regression coefficient suggested that direct subjective norm (β = 0.27), was the main predictor of intention to be screened followed by direct attitude (β = 0.25), past experience of screening (β = 0.11), and positive HIV status (β = 0.10). The variance explained in intention for cervical cancer screening from all predictors was 38.51%.

For a unit increase in the score of the direct subjective norm, the intention to receive cervical cancer screening was increased by 0.09 provided that other variables were kept constant. Similarly, a unit increase in the score of the direct attitude resulted in increased intention by 0.09 other variables are kept constant. Compared to HIV-negative commercial sex workers, intention to receive cervical cancer screening increased by 0.26 times among HIV-positive women. Similarly, women who had an experience of being screened for cervical cancer increases their intention by 0.28 times to receive cervical cancer screening services than their counterparts (Table 5).

**Table 5:** Factors associated with intention to receive cervical cancer screening among CSWs in Gondar city, 2021 (*n* = 393)

Variables	Unstandardized beta(B)	Standardized beta (β)	95% CI of B
Constant ^a^	0.27		(-0.39, 0.93)
Direct attitude ^a^	0.09*	0.25	(0.04, 0.13)
Direct Subjective norm#	0.09*	0.27	(0.05, 0.13)
Direct perceived behavioral control ^a^	0.04	0.12	(-0.003, 0.10)
Age ^a^	-0.01	-0.04	(-0.02,0.01)
History of STI			
No (ref)			
Yes	-0.16	-0.08	(-0.33, 0.01)
Past screening experience			
No (ref)			
Yes	0.28*	0.11	(0.09, 0.47)
HIV status			
Negative(ref)			
Positive	0.26*	0.10	(0.06, 0.46)
Unknown	0.08	0.02	(-0.21, 0.38)
Knowledge			
Poor (ref)			
Moderate	0.02	0.01	(-0.19, 0.23)
Educational status			
No formal education(ref)			
Primary education	-0.06	0.03	(-0.24, 0.12)
Secondary education and above	0.01	0.01	(-0.21, 0.24)

^a^ continues variable *p < 0.05, ref. = reference

## Discussion

This study assessed the intention to receive cervical cancer screening service and its predictors among CSWs in Gondar city. The median intention to receive the screening service was 4 with an IQR of (3 to 4). Direct subjective norm, direct attitude, positive HIV status and past experience of cervical cancer screening was the predictor of intention for cervical cancer screening.

This study revealed that the TPB variables explained 38.51% of the variance in intention to receive cervical cancer screening. The finding was similar to a meta-analysis of TPB accounted for 39% of the variance in intentions [24]. But, this finding is lower than the study conducted among, in which the model explained 65% of the variance in intention [29]. This may be because of the analysis method used in the study is different which is structural equation modelling which accounts for errors in the model. This variance is higher than other studies done in Latina (27.6%) [25] and Thailand (28.1%). This discrepancy may be attributed to the difference in how the intention to receive cervical cancer was measured. In the current study, the intention was measured by four items. However, the study done in Latina used single yes/no questions to measure intention. The variance explained in intention by the model was good enough to suggest an approach for designing health program interventions to increase cervical cancer screening intentions. This implies that future educational interventions that target commercial sex workers would be successful if it is based on theory of planned behaviour variables.

In this study, the median intention to receive cervical cancer screening was 4 (interquartile range 3–4.25). This finding is consistent with a study done in the United States among African Americans (4.38 ± 0.7), Latina women (4.57 ± 0.73) [34], in Jimma (3.63 ± 1.0) [28], and Bahr Dar city (3.5 ± 0.67) [26]. But it is higher than a study done in Debre Birhan (2.65) [42]. This may be due to the difference in the population where in Debre Birhan among women in the general population whose age groups are above thirteen. The other difference may be the period (nowadays it is given more emphasis for cervical cancer screening).

In this study, the direct subjective norm was the strongest predictor of intention to be screened for cervical cancer. This result is consistent with studies done in Singapore and Iran where the subjective norm is the main predictor of intention [43–45],and a study done on low-income women [46]. This means respondents who have high support and approval from the important others (volunteers, health professionals, and their friends) increase their intention to receive cervical cancer screening. Thus, interventions should focus on strengthening friends and organizing volunteers than focusing on the individual alone. But this finding was contrary to a study done in Canada where the subjective norm is not a predictor of intention [36, 47]. This may be due to socio-cultural differences among the participants in this study and the industrialized country, and individual sociality differs across the country.

Direct attitude is the second strongest predictor of intention. This study finding is in line with a study done in Bahrdar city [26], a study done on low-income women[46], a study in Iran[45] and a study in British[48]. This means that respondents having the belief that cervical cancer screening can (detect the disease at an early stage, reduce the risk of getting cervical cancer, and be useful to get treatment early) increases their intention to receive cervical cancer screening. However, inconsistent with studies done among African American and Latina women [34] and a study done in Canada [49]. This may be due to the difference in the context of the study area, and sociodemographic factors.

Past cervical cancer screening experience is a significant predictor of intention to be screened for cervical cancer. Respondents' past behavior of screening does have high intentions than those who have not such experiences. Similarly, a meta-analysis shows that past behavior is a predictor of intention after taking into account on theory of planned behavior variables [50]. This is also supported by studies done in Bahr Dar, and Latinas-America [25, 26]. On the other hand, being HIV positive is another predictor of intention to be screened for cervical cancer. Respondents who report positive HIV status do have high intentions as compared with those who report negative HIV status. This may be due to their past experience and follow up given from different health organizations. Those HIV positives started antiretroviral therapy and have a follow-up in different health institutions so that they may be told to have cervical screening. This implies that interventions given for HIV-positive individuals should be extended to HIV-negative individuals to increase their intention to receive cervical cancer screening.

In this study perceived behavioral control was not a significant predictor of intention. This is in line with a study done in London where TRA can be a substitute for TPB [51]. But this was contrary across different studies done in the globe where perceived behavioral control is one of the predictors of intention [25–27]. The relative importance of attitude, subjective norm, and perceived behavioral control in the prediction intention are expected to vary across behaviors and situations [23]. In situations where attitude or subjective norm is powerful in influencing intention, perceived behavioral control may be less predictor of intention[23]. This implies that TRA was useful in explaining behaviors under a person’s willful (volitional) control.

Sociodemographic variables were not statistically significant predictors of intention. This was in line with a study done in southern Ethiopia [27]. But this was contrary to other studies done in Iran where age is a predictor of intention to get a pap smear test [44], and in Canada, higher education is a significant predictor of intention [47]. This may be due to differences in the context of the study area, and the difference in sociodemographic factors.

### Strengths and limitations of the study

Regarding the strength of the study, it touched on an area that has been overlooked; especially there is interruption of cervical screening during the pandemic of COVID-19[52].

However, it is not free of limitations; we have used convenience sampling, so that the findings may not be generalized for all source populations. During face-to-face interviews, there may be an introduction of social desirability bias. Bear in mind that this research only measures their intention without accounting for their actual behavior to be screened, so it did not assess how much of the intended respondents will have the actual behavior (cervical cancer screening). The other limitation of this study is related to the theory of planned behavior that does not account for environmental factors[53].

## Conclusions

In the present study, TPB explained adequate variance in intention to receive cervical cancer screening. The median intention to use cervical cancer screening was high. We found that behavioral intention to be screened for cervical cancer was a function of attitude, subjective norm, past experience, and positive HIV status. Subjective norm was the strongest predictor of intention to receive cervical cancer screening. Healthcare planners increase participants’ rates of cervical cancer screening intention by developing projects and programs considering the influence of subjective norms that is the significant others including friends, and volunteers. Preparing training by focusing on awareness creation activities to increase the importance of having cervical screening to form a favorable attitude towards screening could be beneficial. Longitudinal research is needed to see how behavioral intention is transformed into cervical cancer screening behavior.

## Data Availability

The datasets used and analyzed during this study is freely available: https://www.kaggle.com/datasets/habitubirhaneshetu/csws-intention-to-cca-screening.

## Abbreviations

AIDS: Acquired Immune Deficiency Syndrome
CI: Confidence Interval
CIN: Cervical Intraepithelial Neoplasia
CSWs: Commercial Sex Workers;
HIV: Human Immune Deficiency Virus
HPV: Human Papilloma Virus
IQR: Interquartile Ranges; Pap: Papanicolaou
